# The Influence of Hydraulic Ankles and Microprocessor-control on the Biomechanics of Trans-tibial Amputees During Quiet Standing on a 5° Slope

**DOI:** 10.33137/cpoj.v2i2.33517

**Published:** 2020-02-24

**Authors:** M McGrath, KC Davies, P Laszczak, B Rek, J McCarthy, S Zahedi, D Moser

**Affiliations:** Blatchford Group, Unit D Antura, Bond Close, Basingstoke, RG24 8PZ, UK.

**Keywords:** Prosthesis, Transtibial Amputees, Gait Analysis, Kinematic, Kinetic, Amputation, Microprocessor foot, Hydraulic ankle, Slope, Symmetry, Standing balance

## Abstract

**BACKGROUND:**

Lower limb amputees have a high incidence of comorbidities, such as osteoarthritis, which are believed to be caused by kinetic asymmetries. A lack of prosthetic adaptation to different terrains requires kinematic compensations, which may influence these asymmetries.

**METHOD:**

Six SIGAM grade E-F trans-tibial amputees (one bilateral) wore motion capture markers while standing on force plates, facing down a 5° slope. The participants were tested under three prosthetic conditions; a fixed attachment foot (FIX), a hydraulic ankle (HYD) and a microprocessor foot with a ‘standing support’ mode (MPF). The resultant ground reaction force (GRF) and support moment for prosthetic and sound limbs were chosen as outcome measures. These were compared between prosthetic conditions and to previously captured able-bodied control data.

**RESULTS:**

The distribution of GRF between sound and prosthetic limbs was not significantly affected by foot type. However, the MPF condition required fewer kinematic compensations, leading to a reduction in sound side support moment of 59% (p=0.001) and prosthetic side support moment of 43% (p=0.02) compared to FIX. For the bilateral participant, only the MPF positioned the GRF vector anterior to the knees, reducing the demand on the residual joints to maintain posture.

**CONCLUSIONS:**

For trans-tibial amputees, loading on lower limb joints is affected by prosthetic foot technology, due to the kinematic compensations required for slope adaptation. MPFs with ‘standing support’ might be considered reasonable and necessary for bilateral amputees, or amputees with stability problems due to the reduced biomechanical compensations evident.

## INTRODUCTION

Musculoskeletal health problems are prevalent among lower limb amputees.^1–4^ The consensus among biomechanists is that the inherent asymmetry of the body, along with reduced confidence and proprioception on the prosthetic side, leads to an unequal distribution of limb loading between the two limbs.^2,3,5^ Excessive dependence on the sound limb for support can have a degenerative effect on the joints. Osteoarthritis (OA) is common, with studies reporting incidences in up to 41% of trans-tibial amputee^4,5^ (TTA) community. Most often, OA presents in the sound knee joint, affecting between 12-66% of all lower limb amputees,^2,3,6^ however up to 23% are also affected at the sound hip.^2,3^ There are also consequences for the residual side through reduced loading. Osteoporosis (OP) and osteopenia have been reported to affect the residual limbs of approximately 90% of people with lower limb amputation.^3,4^ One research study found a mean reduction in bone density of 15% across lower limb amputees, compared to the intact limb.^2^

Another consequence of loading asymmetry is low back pain (LBP). The rate of occurrence has been reported to be as high as 48-71% in the lower limb amputee population^1,3,7–9^ as a whole and 62% for TTAs,^10^ in particular. This figure is approximately double the estimated 28-30% of the general population that are affected by back pain.^9,11^ Research has also highlighted how quickly this problem can develop, with 60% of amputees reporting moderate to extreme back pain occurring within the first two years after amputation.^10^

The alignment of the lower limb prosthesis is one factor key to achieving a ‘close-to-normal’ posture and gait with even distribution of loads between prosthetic and sound limbs. The position and movement of the body’s centre-of- mass (COM), relative to the positions of the lower limb joints, influences the forces and moments acting at those joints.^12,13^ This in turn affects the muscular effort required to provide support.^14^ Typically, the alignment process within a clinic focuses on level ground ambulation. However, when walking or standing on uneven or sloped surfaces, the relative position of the body’s COM to the lower limb joints changes and thus the alignment of a prosthesis may become sub-optimal.^15–18^

Part of the problem may be the prosthetic device. In conventional, energy-storing-and-return (ESR) feet that have a fixed attachment to the distal end of the prosthetic pylon, plantarflexion and dorsiflexion of the foot are achieved through deformation of the foot keel, which is often constructed of elastic elements and structures. If the foot cannot comply with sloped surfaces, compensatory movement of other joints may be required to ensure the foot is flat on the ground.^19^ This is due to the reaction forces that are induced and act on the body as the keel deflects from a neutral unloaded condition. For unilateral TTAs, this often presents as increased residual knee flexion.^20,21^ This action puts the body’s COM behind the knee joint centre, creating a flexion moment, which may require greater effort to resist. Not only does this increase the day-to-day energy consumption of the user, but it also has negative implications on their stability and sound limb dependence. Hydraulic ankles, which produce a viscoelastic response when loaded, allow a degree of damped movement proximal to the foot springs, ‘self-aligning’ with the ground and helping to maintain the body’s COM position relative to the lower limb joints.^19,22^ This action minimises undesirable biased forces and moments acting on the limb, thus it may also reduce the compensations needed to enable effective standing on uneven surfaces.^19^

Previous work has used the ground reaction force (GRF) to determine the inter-limb load distribution during transfemoral amputee (TFA) standing tests.^19^ This approach, however, does not consider the loading at individual joints, which could provide greater insight into which joints are most at risk of OA development. Furthermore, since possible compensation strategies may vary between amputees,^19^ Winter’s concept of support moment^23^ may provide a more comprehensive and universal metric by which to measure the demand on a given limb to provide support. This is defined as the sum of the moments acting at the ankle, knee and hip, where extension moments make up the positive contribution to support and flexion moments provide a negative one.^23^

This study expands on the previous work^19^ to focus on TTAs and, in particular, how differences in prosthetic foot and ankle technology can influence the way in which they stand on non-level ground. Two hypotheses were tested. The first was that TTAs would present with asymmetry in GRF distribution when using a fixed attachment foot, which would be reduced when using adaptive feet. The second hypothesis was that the fixed attachment foot would lead to increased lower limb joint moments, compared to adaptive feet.

## METHODOLOGY

### Prosthetic devices

Three different prosthetic ankle/foot devices were evaluated in this study, each of which uses different prosthetic technology. The first was Esprit^i^ (FIX – Blatchford, Hampshire, UK), which is an ESR foot with a fixed attachment to the distal end of the prosthetic pylon. The second device was Echelon^ii^ (HYD – Blatchford, Hampshire, UK), which shares a common geometry with Esprit but with a hydraulic ‘ankle’ unit attached proximally. Plantarflexion and dorsiflexion of the foot are achieved through a combination of rotation of the hydraulic unit (allowing for 9° of damped movement) and the deformation of foot springs. The final device was Elan^iii^ (MPF – Blatchford, Hampshire, UK), with a hydraulic system similar to Echelon, which includes microprocessor- control. The hydraulic unit provides damped ankle flexion adapting in real time to slopes and changes in speed but when the device detects that the user is standing still, the hydraulic resistances to movement in the plantarflexion and dorsiflexion directions are increased to a high level. This change is intended to provide both ground adaptation and extra support when standing. The high resistance is such that it permits small natural alignment adjustments but does not ‘lock’ the ankle in a fixed position, which may or may not be optimal.

### Participants

Six TTA participants volunteered for the study, the details of whom are listed in Table 1. Verbal participant information was given and signed consent was provided by each participant. An ethics review of the study followed the tenets of the Declaration of Helsinki and was approved by the institutional ethics review board. Each person was aged 18 or over and, at the time of testing, their residual limbs were in good health, free from infection or skin conditions. A consultant prosthetist determined a SIGAM mobility grade E or higher for all participants, meaning they were capable of negotiating environmental barriers, such as sloped ground and ramps, with no other walking aids. Each of the participants had experience using both fixed attachment and hydraulic ankle/foot devices. They had all initially been prescribed fixed attachment feet immediately post amputation and three (TT1, TT3 and TT4) still used this type of device as a running limb. At the time of testing, all participants had been using a hydraulic ankle or a microprocessor-controlled hydraulic ankle as their habitual, everyday device for at least 12 months.

**Table 1:** Characteristics of the participants. Please note EchelonVT is a hydraulic ankle with an in-built vertical shock pylon/torsion adaptor and EchelonVAC is a hydraulic ankle with an in-built mechanism for generating elevated vacuum suspension.

Subject	Gender	SIGAM grade	Age (years)	Mass (kg)	Height (m)	Prosthetic limb	Habitual prosthesis
TT1	Male	F	42	51	1.65	Right	EchelonVT
TT2	Male	F	24	60	1.70	Right	Elan
TT3	Male	F	38	92	1.83	Right	EchelonVT
TT4	Male	F	53	65	1.78	Left	EchelonVAC
TT5	Male	F	45	92	1.77	Right	Elan
TT6	Female	E	36	55	1.75	Both	2x Elan

The data gathered during this study were compared to the same measurements gathered from a group of able-bodied control participants in a previous study^19^ (27.4±2.9 years, 66.8±10.3 kg).

### Gait lab setup

A motion capture system was used to track the movements of participants (Codamotion, Charnwood Dynamics, Leicestershire, UK). This system uses active marker clusters, two three-dimensional infra-red cameras and two force plates (Kistler Group, Winterthur, Switzerland) positioned side by side on a 5° slope. The cameras collected data at a frequency of 200Hz, while the force plate acquisition frequency was 500Hz. Body segment tracking and definitions of virtual markers were done using a conventional six-degree-of-freedom (6DoF) marker model^24^ similar to that used in a previous study.^19^ The marker model was designed for able-bodied participants and required virtual markers at the medial and lateral malleoli. On the prosthetic limb, these were defined at the pivot point of the hydraulic units for the HYD and MPF devices. The similar geometry of the FIX device meant that corresponding locations could be approximated when that device was worn.^19^

### Data collection

Participants were asked to wear tight fitting shorts and t-shirts to permit the accurate positioning of markers, reduce marker occlusions and minimise movement artefact. Each wore regular trainers and the same footwear was used for each prosthetic device tested.

The testing protocol was based on that used in a previous study.^19^ Participants stood facing down a 5° slope and, when instructed, stepped forwards, placing one foot on each of the two adjacent force plates. Once on the force plates they were instructed to stand as naturally as possible. Multiple trials (minimum of three) were used to measure at least 30 seconds of standing per participant. This meant that no single prosthetic device was detrimentally affected by initial foot positioning and steadying when a participant first stepped onto the force plates.

Each participant performed the testing protocol with each of the three prosthetic ankle/foot devices. Each device was fitted and aligned by the same experienced senior prosthetist and the order in which they were tested was randomised. Before data collection began with a new prosthetic device, the participants were given 30 minutes to acclimatise to the new foot. Since each participant was already experienced with both fixed attachment and hydraulic prostheses, this time was deemed sufficient. Regardless, testing would only proceed once both the participant and prosthetist were satisfied that they were capable of performing the protocol safely.

### Data processing and analysis

In order to ensure only quiet standing was analysed, the actions of stepping on and off of the force plates needed to be excluded. The final 3 seconds of each trial were rejected and the preceding 10 seconds were extracted and used in the final analysis.

All kinetic parameters were normalised by the participant’s mass so that data were comparable between participants. All data were processed and analysed with Visual3D v6 x64 biomechanics analysis software (C-motion Inc., Germantown, MD, USA).

### Statistical analysis

The data were assessed for normality using Shapiro-Wilk tests and for homogeneity of variance using Fligner-Killeen tests. For normally distributed data, a one-way analysis of variance (ANOVA) was used to identify statistically significant differences between the different prosthetic ankle/foot technologies and post-hoc Tukey tests were performed for pairwise comparisons. For non-normally distributed data, or for groups with heterogeneity of variances, Kruskal-Wallis tests were followed by post-hoc Dunn tests. Statistical significance was defined as p<0.05. All statistical tests were performed using R v3.3.3 (The R Foundation, Vienna, Austria).

## RESULTS

### Kinematic compensations

**Figure 1:**
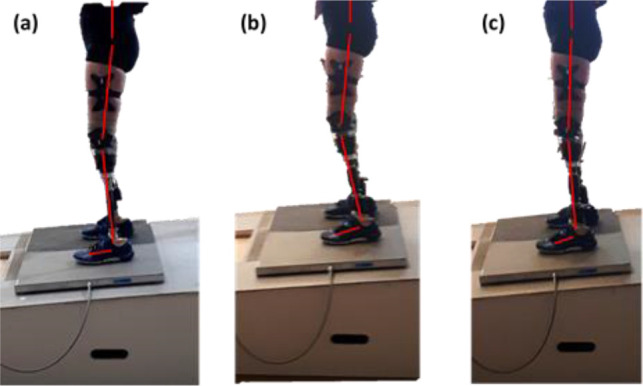
The kinematic strategies of the bilateral trans-tibial amputee participant using (a) FIX, (b) HYD and (c) MPF.

There were no significant differences between the mean joint angles for unilateral participants between prosthetic conditions. The bilateral amputee, however, did present with clear kinematic compensations (Figure 1). The FIX condition required knee flexion in order to achieve foot-flat. Interestingly, for the HYD condition, knee flexion increased further (not significant) as the participant ‘rested’ on the mechanical dorsiflexion stops at the limit of the hydraulic range. The MPF condition allowed knee flexion to be reduced to a more upright posture (p=0.002 compared to FIX).

### Kinetics (bodyweight distribution)

There were no statistically significant differences in the resultant GRF, or any of the three axial components, between the prosthetic conditions for the unilateral amputees. Differences in the mean prosthetic and sound values were all less than 3%. The prosthetic condition did not significantly influence GRF for the bilateral amputee either.

### Kinetics (joint moments)

**Figure 2:**
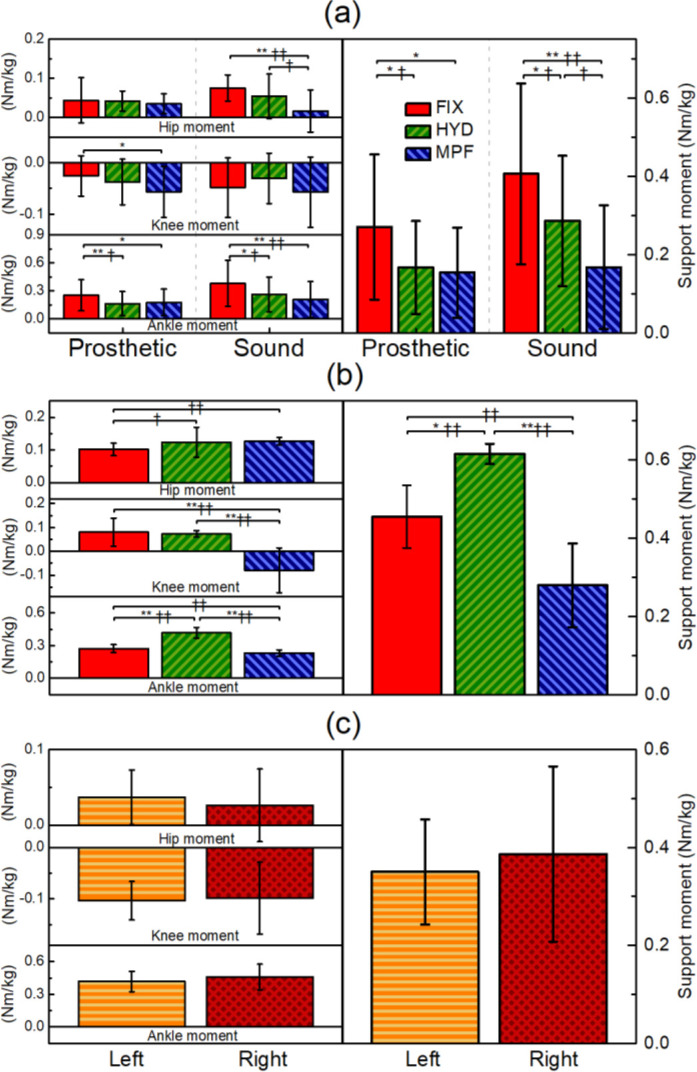
The relative contributions of ankle, knee and hip moment to the total support moment for (a) unilateral trans-tibial amputees, (b) a bilateral trans-tibial amputee and (c) able-bodied control participants. Asterisks indicate statistical significance: *p<0.05, **p<0.01. Obelisks indicate effect size changes: †|d|>0.5, ††|d|>0.8.

Both prosthetic and sound support moments were significantly affected by prosthetic condition for the unilateral group (Figure 2a). Post hoc testing showed that, compared to FIX (0.27 ± 0.19 Nm/kg), both HYD (0.17 ± 0.12 Nm/kg, p=0.038) and MPF (0.15 ± 0.12 Nm/kg, p=0.020) reduced prosthetic side support moment. Sound side support moment was also reduced by HYD (0.29 ± 0.17 Nm/kg) compared to FIX (0.41 ± 0.23 Nm/kg, p=0.045). MPF reduced sound side support moment further (0.17 ± 0.16 Nm/kg), which was statistically significant compared to FIX (p=0.001) but not compared to HYD.

These changes were mostly due to different ankle kinetics. The ankle moments for FIX (0.25 ± 0.17 Nm/kg prosthetic; 0.38 ± 0.25 Nm/kg sound) were significantly decreased by HYD (0.16 ± 0.13 Nm/kg prosthetic, p=0.009; 0.26 ± 0.19 Nm/kg sound, p=0.034) and by MPF (0.18 ± 0.14 Nm/kg prosthetic, p=0.042; 0.21 ± 0.19 Nm/kg sound, p=0.004). Another statistically significant change was the reduction of sound hip moment by MPF (0.02 ± 0.05 Nm/kg) compared to FIX (0.08 ± 0.03 Nm/kg, p<0.001). When MPF was compared to HYD (0.06 ± 0.06 Nm/kg), the reduction approached significance (p=0.06).

For the bilateral amputee, support moment for HYD (0.62 ± 0.03 Nm/kg) was significantly higher than both FIX (0.46 ± 0.08 Nm/kg, p=0.002) and MPF (0.28 ± 0.11 Nm/kg, p<0.001). MPF significantly reduced knee moments (-0.08 ± 0.09 Nm/kg) compared to both other conditions (FIX: 0.08 ± 0.06 Nm/kg, p=0.003, HYD: 0.078 ± 0.01 Nm/kg, p=0.002).

## DISCUSSION

The results of this study disproved the first hypothesis that TTAs would present with GRF distribution asymmetry when standing on a slope with a fixed attachment foot. However, the second hypothesis that the fixed attachment foot would increase joint moments, compared to adaptive feet, was found to be true.

The observation that the TTAs in this study did not display any obvious inter-limb GRF asymmetry for any of the prosthetic technologies is in contrast to the findings of the previous research with TFAs,^19^ suggesting that the participants had the confidence to load their prosthetic limb. This, in the long-term, may help to reduce the likelihood of OP^25^ or LBP^1^ development. Furthermore, again in contrast to the TFA participants,^19^ unilateral TTAs did not present any significant differences in mean joint angles between prosthetic conditions. Upon closer inspection, this observation could be attributed to the inter-participant variation and different strategies used to adapt their limbs to the gradient, some with knee flexion, some with hip flexion. This led to broad standard deviations, masking any trends. Additionally, compared to TFAs, it is possible that the extra control allowed by the residual knee joint meant that a foot-flat position could be achieved through greater foot spring deflection, with a reduced amount of knee flexion.

These observations perhaps highlight the advantage of using the support moment metric for standing biomechanics and prosthetic alignment. The concept of support moment was devised by Winter^23^ as a metric for use in gait analysis to show the bodyweight support provided by a limb as a whole. This research used the same method of calculation applied to quiet standing. The advantage of this approach is that it gives greater insight into the demand on the joints and how it is affected by changing test conditions, rather than only the weight-bearing load. Additionally, this metric is not sensitive to the compensation strategy used so it is not obscured by inter-participant variability. For the unilateral TTAs, statistically significant changes were observed in both prosthetic and sound support moments. This shows that even though the participants were applying equal loads to their limbs, there was still an adverse effect on their sound side joints, which could be a risk factor in OA development.^26,27^ In the case of unilateral amputees, for example, the FIX condition presented a significantly higher demand on the sound ankle (p=0.004) and hip (p<0.001), compared to MPF. Consequently, the MPF presented the best scenario for the sound joints, as participants were able to align their joints to minimise the moments acting about them.

In a modelling analysis of quiet standing, Winter highlighted that in the lower limbs, the ankle joint provided the greatest contribution to posture and balance.^28^ This was confirmed by the able-bodied control participants in this study where, of the individual joints, the greatest contribution to support was found at the ankle (Figure 2c). This trend was also present in the sound limbs of the unilateral amputees, highlighting the potential benefits of a prosthetic technology that could reduce the demand on the ankle joint while maintaining a comfortable standing posture.

These observations are what makes the bilateral TTA participant such an interesting case study. The lack of any sound ankle – integral to maintaining posture^28^ – shifts the reliance to the knees or, indeed, the prosthetic technology. In this case, knee moments were affected by prosthetic condition. MPF was the only condition to produce negative knee moments. This indicated that the ground reaction vector had passed anterior to the knee joints. When prosthetists align devices in the clinic, this is one of their goals to achieve adequate balance with minimal muscular demand. The ability of a prosthetic ankle to adapt to changing gradients in this way is invaluable for a bilateral amputee, but extra prosthetic technology is required to compensate for the lack of a sound ankle. The HYD and MPF devices in this study have the same hydraulic range but, for the bilateral participant, increased knee flexion was only observed during the HYD condition. This was because the MPF devices provided “standing support”; initially adapting to the slope before increasing the resistance to ‘ankle’ movement when the sensors detected that the user was standing still. This held the MPF devices well-aligned, shifting the knee moment trend in-line with that observed for the able-bodied participants.

Other work has sought to compare different types of prosthetic feet when standing on slopes, but the gradients used were higher than that in the current study (7° slope,^20^ 10° slope^21^ and 15° slope,^29^ respectively) so direct, quantifiable comparisons are challenging. However, similar trends were reported for the comparison of fixed attachment feet to feet with adaptive ankles. Reduced residual knee flexion was observed when using the adaptive feet, compared to fixed,^20,21^ affecting joint moments. Ernst et al.^21^ also noted the different strategies employed by participants to adapt to the slope and how this influences the variability of the measurements.

It is worth acknowledging that the protocol of this study might have influenced the findings to some degree. Software limitations meant that recordings could only be performed for relatively short intervals. Future work might ask participants to stand for longer time periods, recording short intervals throughout that longer period. This would highlight whether differences between prosthetic conditions become more substantial as the participants become more fatigued.

## CONCLUSION

This study has shown that unilateral TTAs are able to maintain approximate weight-bearing symmetry between their prosthetic and sound limbs while standing on sloped ground. However, the demand that is placed on their joints is dependent upon the ease in which they are able to maintain an upright posture. Hydraulic ankles allow self-alignment, resulting in fewer kinematic compensations and reducing the moments on the sound joints. For bilateral TTAs, the combination of ankle adaptation and standing support provided by the MPF, represented the only condition under which the ground reaction vector was anterior to the knee joints. This suggests that MPF technology is particularly important for bilateral amputees, in order to protect the joints against excessive demand and the development of OA.

## DECLARATION OF CONFLICTING INTERESTS

The authors are full time employees of the manufacturer of the prosthetic devices examined in this study.

## ETHICAL APPROVAL

An ethics review of the study followed the tenets of the Declaration of Helsinki and was approved by the institutional ethics review board.

## AUTHOR CONTRIBUTION

- **Michael McGrath,** Conceptualization, data collection, data analysis, manuscript preparation, review and editing.
- **Katherine C. Davies,** Manuscript preparation, review and editing.
- **Piotr Laszczak,** Data analysis, manuscript review and editing.
- **Beata Rek,** Data analysis, manuscript review and editing.
- **Joe McCarthy,** Conceptualization, data collection, manuscript review and editing.
- **Saeed Zahedi,** Conceptualization, manuscript review and editing.
- **David Moser,** Conceptualization, manuscript review and editing.

## SOURCES OF SUPPORT

Blatchford provided financial support and prosthetic devices for this study.

## MANUFACTURERS’ DOCUMENTATION

i http://www.blatchfordus.com/products/esprit

ii http://www.blatchfordus.com/products/echelon

iii http://www.blatchfordus.com/products/elan
